# Novel MCM-41 Supported Dicationic Imidazolium Ionic Liquids Catalyzed Greener and Efficient Regioselective Synthesis of 2-Oxazolidinones from Aziridines and Carbon Dioxide

**DOI:** 10.3390/molecules28010242

**Published:** 2022-12-28

**Authors:** Yulin Hu, Lili Yang, Xiaobing Liu

**Affiliations:** 1 College of Chemistry and Chemical Engineering, Anshun University, Anshun 561000, China; 2 College of Chemistry and Chemical Engineering, Jinggangshan University, Ji’an 343009, China

**Keywords:** supported ionic liquid, CO_2_ conversion, aziridines, 2-oxazolidinones, sustainability

## Abstract

A type of MCM-41 supported dicationic imidazolium ionic liquid nanocatalyst has been synthesized and found to be competent for the synthesis of 2-oxazolidinones through the sustainable chemical conversion of CO_2_ with aziridines. It was shown that the highest efficiency was achieved in the cycloaddition of a series of aziridines and CO_2_ in the presence of a catalytic amount of the solid catalyst MCM-41@ILLaCl_4_ under mild conditions. Merits of this meticulously designed protocol are the use of a novel supported ionic liquid catalyst, the easy work-up process, good to excellent yields, a short reaction time, and purification without column chromatography. Overall, the present protocol of synthesizing 2-oxazolidinones under cocatalyst- and solvent-free conditions using MCM-41@ILLaCl_4_ is promising for industrial applications.

## 1. Introduction

The capture and transformation of CO_2_ into valuable chemicals is considered as one of the ideal solutions to address issues concerning the crisis of fossil fuels and CO_2_ emissions [1,2,3,4]. As a promising strategy for CO_2_ chemical utilization, the cycloaddition reaction of CO_2_ with aziridines is one of the most atomeconomic, sustainable, and green approaches to the conversion and storage of carbon dioxide. 2-Oxazolidinones are an important class of N-heterocyclic compounds and have been widely used as intermediates to produce useful pharmaceuticals, auxiliaries, cosmetics and pigments [5,6,7,8]. Considering the importance of 2-oxazolidinones and 100% atomic economy, numerous catalytic strategies have been developed towards the cycloaddition reaction of carbon dioxide with aziridines for the synthesis of 2-oxazolidinones, such as organocatalysts [9,10], TPPH_2_@SBA-15 [11], confined nanospaces of hierarchical porous silica [12], metal complexes [13,14,15], catechol porphyrin COF [16], metal–organic frameworks [17,18], HKUST-1/TBAB [19], PEG_6000_(NBu_3_Br)_2_ [20] and others [21,22,23]. Although these reported catalytic processes are quite useful, most of these processes has one or more limitations, such as a difficult work-up process, the use of toxic solvents and expensive catalysts, difficulty in the recycling of the catalyst, drastic reaction conditions and environmental contamination. Thus, to overcome these disadvantages, the development of simple, efficient and environmentally friendly catalytic systems for the chemical fixation of CO_2_ into 2-oxazolidinones via sustainable cycloaddition with aziridines remains a very important research topic.

**Scheme 1:**
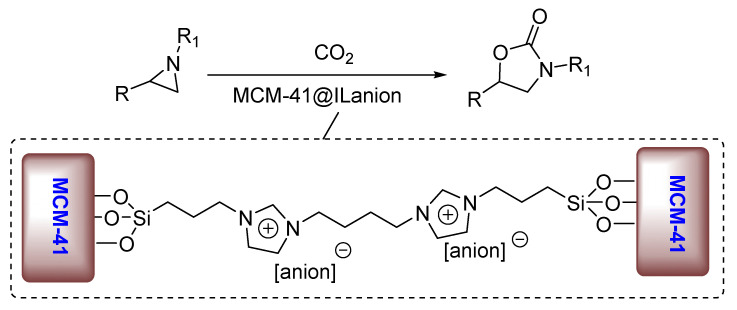
Schematic diagram for catalytic synthesis of 2-oxazolidinones from CO_2_ and aziridines.

Ionic liquids (ILs) have been considered as promising and sustainable materials in chemical synthesis and catalysis due to their unique properties of low vapor pressure, non-volatility, an adjustable organization structure, biocompatibility and high stability (thermal and chemical) over conventional organic solvents [24,25,26,27,28]. Several ILs, as catalysts, as reaction media or as both catalysts and reaction media, have been employed in the cycloaddition reaction of carbon dioxide with aziridines for the synthesis of 2-oxazolidinones [29,30]. Although these processes are useful in many aspects, they are limited by the difficulty in the separation, recovery and recyclability of the ionic liquid catalytic system. To solve these shortcomings, the concept of the immobilization of ILs onto solid support materials for the formation of supported ionic liquids can be adopted as an effective strategy to achieve improved catalytic properties and facilitate the separation and recovery of the catalysts [31,32,33,34,35]. Among the preferred solid support materials, mesoporous MCM-41 has drawn much attention because of its advantageous properties, such as high surface area and pore volume, excellent thermal and chemical stability, the profusion of exposed silanol (Si-OH) groups, facile separation from the reaction media, hexagonal array uniform mesopore structures and easy handling and functionalization [36,37,38,39,40]. The above-mentioned properties have made MCM-41 a suitable support for the immobilization of functionalized ILs as it creates a number of IL active sites, which could possess promising properties towards sustainable materials and catalytic synthesis [41,42,43,44,45,46]. These sustainable nanomaterials are widely used in many fields of catalysis and adsorption for their unique properties, including their stable porous structure, large surface area and tunable pore sizes, etc. Prompted by these findings, herein, we decided to prepare a type of MCM-41 supported dicationic imidazolium ionic liquid and evaluated its catalytic performance for the sustainable synthesis of 2-oxazolidinones via the sustainable cycloaddition of CO_2_ with aziridines under mild conditions (Scheme 1). Moreover, the catalyst could be easily reused in successive catalytic runs.

## 2. Results and Discussion

The catalytic activities of the supported ionic liquid nanocomposites were investigated in the model cycloaddition reaction of CO_2_ and 2-methylaziridine, and the results are shown in Table S1 (Supplementary Materials). Initially, the different supported ionic liquid catalysts of MCM-41@ILBF_4_, MCM-41@ILLaCl_4_ and MCM-41@ILCH_3_COO were examined in a model reaction under the same reaction conditions (Table S1, entries 1–3). It was found that the supported ionic liquid MCM-41@ILLaCl_4_ exhibited higher catalytic activity than MCM-41@ILBF_4_ and MCM-41@ILCH_3_COO, and MCM-41@ILLaCl_4_ was the most effective for catalyzing this reaction, affording the target product 5-methyloxazolidin-2-one in a yield of 95% with 99.3% selectivity (Table S1, entry 2), possibly due to its excellent abilities of stabilizing and activating the substrates for this reaction. For a further evaluation of the optimum conditions for the reaction, control experiments with an MCM-41 support or bulk IL catalysts were examined for this reaction (Table S1, entries 4–7). It was found that they were unsuitable catalysts for the reaction, as much lower product yields (15~83%) and selectivity (75.1~94.2%) were obtained. These results indicated that MCM-41@ILLaCl_4_ is an effective catalyst with high catalytic activity for this reaction.

**Figure 1:**
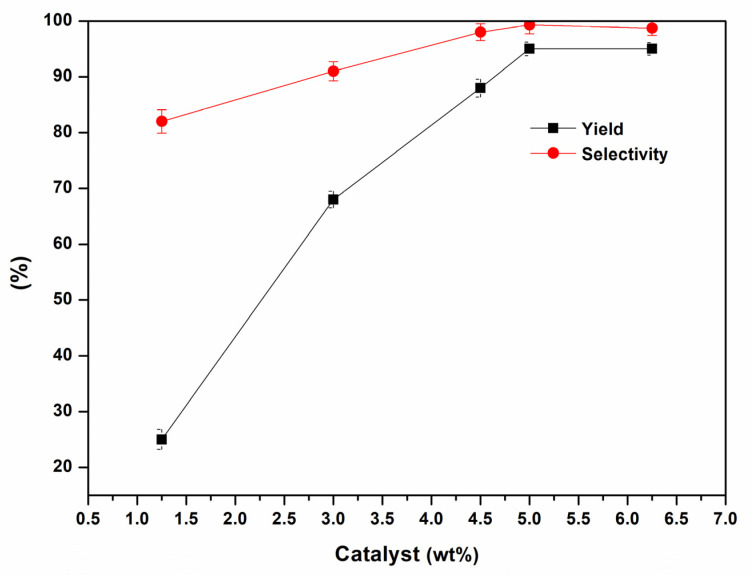
Effect of amount of catalyst on the reaction. Reaction conditions: 2-methylaziridine (10 mmol), CO_2_ (0.7 MPa), MCM-41@ILLaCl_4_, 50 °C, 3 h.

**Figure 2:**
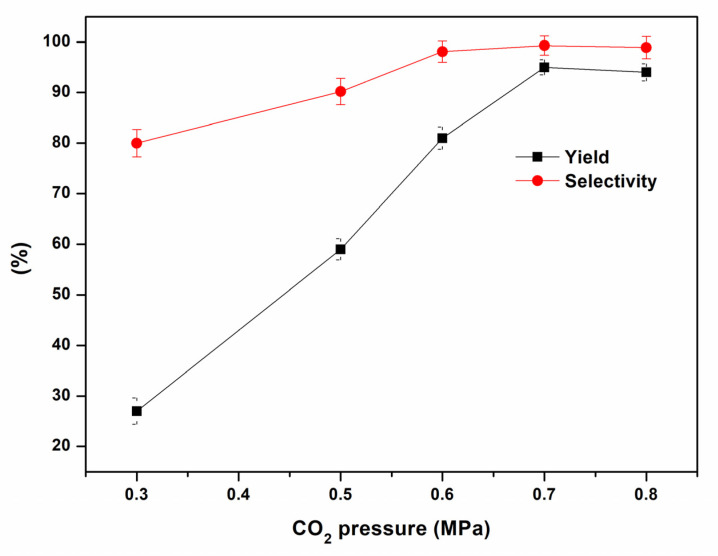
Effect of CO_2_ pressure on the reaction. Reaction conditions: 2-methylaziridine (10 mmol), MCM-41@ILLaCl_4_ (5%, weight percent based on 2-methylaziridine), 50 °C, 3 h.

**Figure 3:**
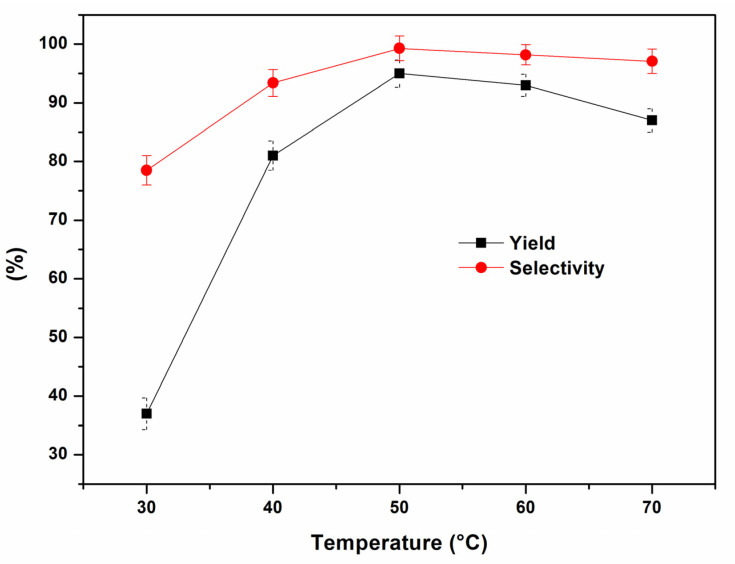
Effect of reaction temperature on the reaction. Reaction conditions: 2-methylaziridine (10 mmol), MCM-41@ILLaCl_4_ (5%, weight percent based on 2-methylaziridine), CO_2_ (0.7 MPa), 3 h.

To obtain further insights into the catalytic reaction conditions of MCM-41@ILLaCl_4_, the influence of the amount of catalyst, CO_2_ pressure and reaction temperature on the cycloaddition were investigated. The cycloaddition reactions were first screened with different amounts of catalysts, and the results achieved are presented in Figure 1. It was observed that the catalytic yield and selectivity significantly increased as the amount of catalyst was increased up to 5% weight percent based on the 2-methylaziridine; however, the catalytic yield and selectivity increased sluggishly upon further increasing the catalyst amount. Thus, the catalyst amount of 5% was optimal for the reaction. The effect of CO_2_ pressure was also screened for the reaction (Figure 2). It was found that the catalytic yield and selectivity significantly increased as the CO_2_ pressure increased from 0.3 MPa to 0.7 MPa; however, no significant enhancement in the yield and selectivity was observed when further increasing the CO_2_ pressure to 0.7 MPa. Thus, the CO_2_ pressure of 0.7 MPa was optimal for the reaction. Moreover, the effect of the reaction temperature was assessed for the cycloaddition reaction (Figure 3). It was found that 50 °C is sufficient to catalyze the cycloaddition reaction efficiently and rapidly, affording the highest yield of 96% and selectivity of 99.5%. Beyond this, the increase in the reaction temperature slowly decreased the yield and selectivity, which was due to the side reactions of isomerization and ring opening, which occurred at higher temperatures (GC analysis). All these results indicated that the optimal temperature for the reaction was 50 °C.

**Figure 4:**
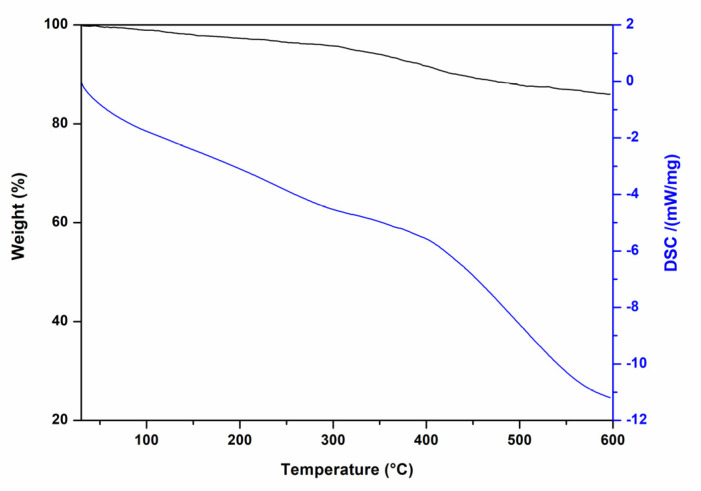
TG analysis of MCM-41@ILLaCl_4_ catalyst.

The thermogravimetric analysis curve plot of the MCM-41@ILLaCl_4_ catalyst is shown in Figure 4. TG/DSC analysis was performed in a nitrogen atmosphere in the temperature range of 25–600 °C. The initial weight loss up to 200 °C was due to the desorption of water and solvent species, which showed 1.97% loss in this region. Further weight loss occurred between 200 °C and 600 °C and was related to the decomposition of the organic IL species, and the observed weight loss was 14.05%. The thermal behavior of MCM-41@ILLaCl_4_ was analyzed by performing a differential scanning calorimetry (DSC) experiment. The corresponding DSC combustion curves revealed two main reaction regions (one small shift of 1.7 mW in heat flow between 25 °C and 200 °C, the other small shift of 7.2 mW in heat flow between 200 °C and 600 °C) for the catalyst. The peaks in the DSC curve also proved this process. Therefore, the catalyst exhibited exceptional thermal stability up to 200 °C, which illustrates the desirable characteristics of the catalyst during the process.

**Figure 5:**
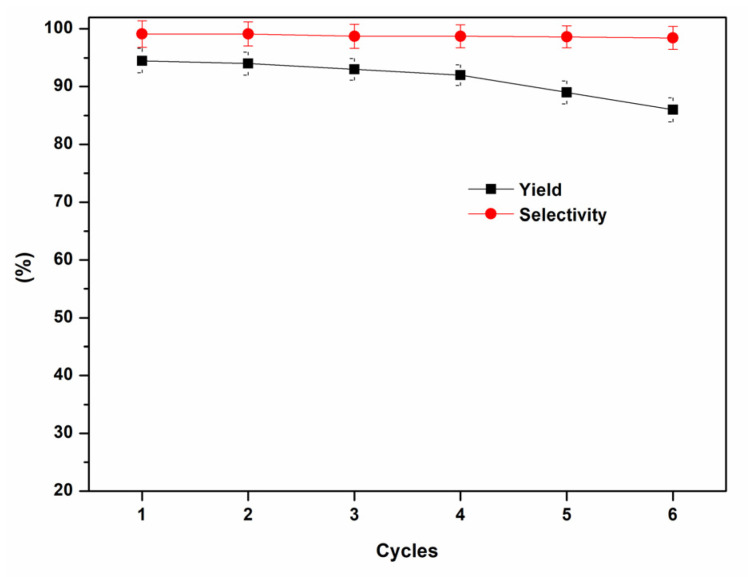
Recyclability study of solid catalyst MCM-41@ILLaCl_4_.

**Figure 6:**
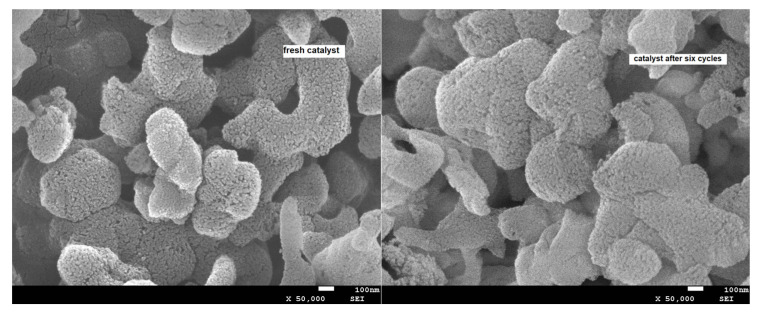
SEM images of catalyst MCM-41@ILLaCl_4_ before and after reaction.

**Figure 7:**
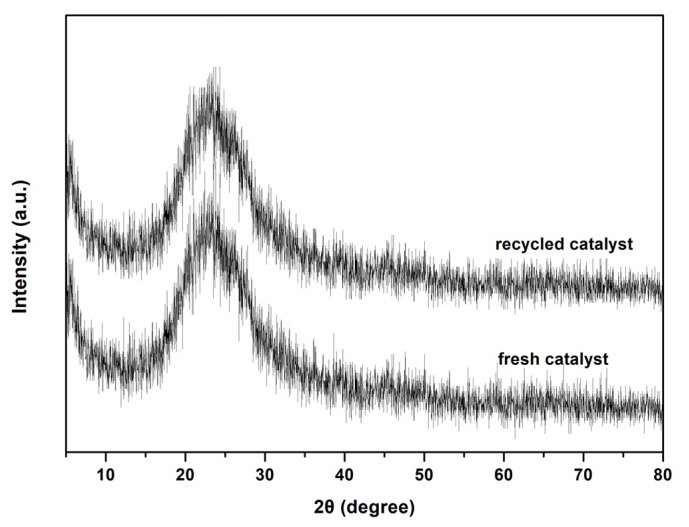
XRD patterns of catalyst MCM-41@ILLaCl_4_ before and after reaction.

The recovery and recyclability of the MCM-41@ILLaCl_4_ catalyst were examined in the benchmark reaction under the optimized conditions (Figure 5). Upon completion of the reaction, the catalyst was easily recovered from the reaction mixture by centrifugation, dried and used directly for the next cycle reaction. The results showed that the catalyst can be effectively utilized for at least six consecutive runs, without a considerable decline in the catalytic activity. Additionally, any morphological changes in the recovered catalyst after six cycles were investigated by SEM (Figure 6), which authenticated that the catalyst was observed to be reusable for at least six runs without any significant change in its mesoporosity. Moreover, the XRD diffractogram of the reused MCM-41@ILLaCl_4_ catalyst after six cycles displayed similar characteristic peaks corresponding to the fresh catalyst (Figure 7), which indicated that the recovered MCM-41@ILLaCl_4_ still had a good mesoporous structure during the course of the reusability studies. These above results evidenced that the chemical environment of the active catalyst could be retained well during the recycling process.

To broaden the potential and general applicability of this protocol, the synthesis of 2-oxazolidinones via the catalytic chemical conversion of CO_2_ and aziridines was examined under the optimized reaction conditions (Table 1). It was observed that a series of aziridines could react with CO_2_ smoothly to provide the desired products in excellent yields of 85~97% and selectivity (≥99%) within 3~5 h. In addition, we observed that the non-substituted groups at the nitrogen atom of the aziridines afforded the desired products in excellent 90~97% yields within 3 h (Table 1, entries 1–5), while the reaction of substituted groups at the nitrogen atom of the aziridines also proceeded smoothly to afford the desired products in high yields of 85~87% after a prolonged reaction time of 5 h (Table 1, entries 6 and 7). These results indicate the excellent catalytic efficiency and good general application of the MCM-41@ILLaCl_4_ catalyst for the synthesis of 2-oxazolidinones.

To evaluate the advantages of the catalytic efficiency of MCM-41@ILLaCl_4_, the research concluded with a comparison of the studied catalyst’s activity with that of previously reported catalysts (Table S2). The presented data show that MCM-41@ILLaCl_4_ is a type of green and suitable catalyst for the efficient chemical conversion of CO_2_ and aziridines into corresponding 2-oxazolidinones under mild conditions. Furthermore, as shown in this table, MCM-41@ILLaCl_4_ could practically completely catalyze the cycloaddition reaction of CO_2_ with aziridines without a solvent and cocatalyst, rendering the MCM-41@ILLaCl_4_ catalyst a potential candidate for practical industrial application.

**Scheme 2:**
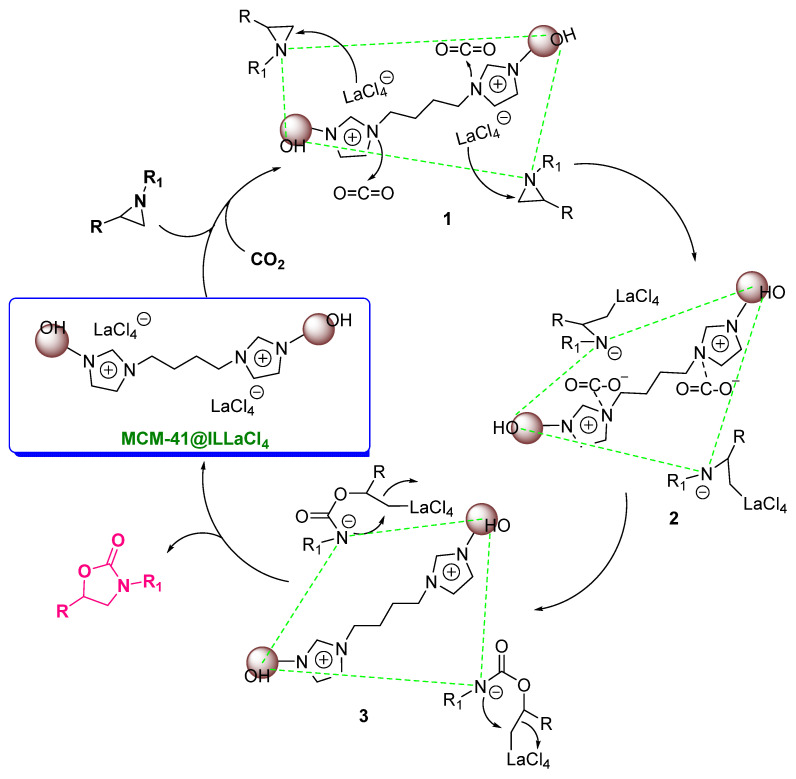
Possible mechanism for the sustainable synthesis of 2-oxazolidinones from CO_2_ and aziridines.

Based on the points mentioned above and reported works [13,14,15,16,17,18,19,29], a possible mechanism for this protocol has been proposed (Scheme 2). Aziridine was initially activated by the Si-OH active sites of MCM-41 via the coordination interaction to form an intermediate **1**, and CO_2_ could be adsorbed and activated by the imidazolium cation to produce carbonate species. Then, the nucleophilic attack of the LaCl_4_ anion on the less sterically hindered C atom of aziridine resulted in the formation of intermediate **2**, which in turn afforded the intermediate **3** via a nucleophilic attack with activated CO_2_. Subsequently, the expected product was afforded by an intermolecular nucleophilic attack (cyclization), together with the regeneration of the synergistic catalyst for the next cycle.

## 3. Conclusions

In conclusion, a type of MCM-41 supported dicationic imidazolium ionic liquid was successfully synthesized through the anchoring of dicationic imidazolium ionic liquids over mesoporous MCM-41, and they were characterized by XRD, SEM, EDX, FT-IR, UV–Vis and BET analysis, provided in the Supplementary Materials. The new ecological nanocatalysts were effectively and heterogeneously employed in the sustainable chemical conversion of CO_2_ and aziridines into 2-oxazolidinones. The catalytic results showed that the MCM-41@ILLaCl_4_ catalyst displayed noteworthy catalytic performance in the cycloaddition of a series of aziridines and CO_2_ to give the desired products with high to excellent yields and selectivity under mild conditions, probably due to the synergetic effects involving the active sites of the ionic liquid and the mesoporous support. The novel recyclable catalyst, mild reaction conditions, excellent yields, shorter reaction times and environmentally benign conditions, avoiding the addition of a cocatalyst or toxic organic solvents, are the noteworthy aspects of the developed protocol. In light of these factors, a greener, more efficient, sustainable, rapid scaffold for the synthesis of 2-oxazolidinones using a novel supported dual imidazolium ionic liquid, MCM-41@ILLaCl_4_, toward the chemical fixation of CO_2_ into valuable chemicals has been demonstrated.

## Figures and Tables

**Table 1:** Catalytic synthesis of 2-oxazolidinones ^a^.

Entry	Aziridine	Product	Time (h)	Yield (%) ^b^	Selectivity (%) ^c^
1	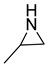	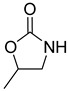	3	95	99.3
2	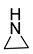	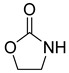	3	97	99.8
3	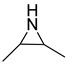	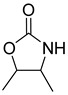	3	92	99.5
4	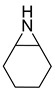	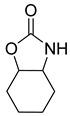	3	90	99.6
5	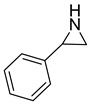	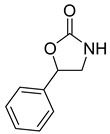	3	94	99.2
6	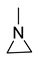	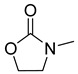	5	87	99
7	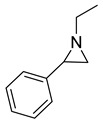	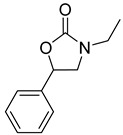	5	85	99.1

^a^ Reaction conditions: aziridine (10 mmol), CO_2_ (0.7 MPa), MCM-41@ILLaCl_4_ (5%, weight percent based on aziridine), 50 °C. ^b^ Isolated yield. ^c^ GC analysis.

## Data Availability

The data are available in the manuscript and Supplementary Materials.

## Supplementary Materials

The following supporting information can be downloaded at: https://www.mdpi.com/article/10.3390/molecules28010242/s1. The experimental section for information of materials, supported IL preparation, catalytic cycloaddition, and characterization are summarized in the supporting information. Scheme S1: Schematic paths for synthesis of supported ionic liquids; Figures S1–S5: XRD diffractograms, SEM images, EDX images, FT-IR spectras, UV–Vis spectras of the supported ILs, respectively; Figure S6: N_2_ adsorption-desorption isotherms and pore size distributions of MCM-41@ILLaCl_4_ and MCM-41; Table S1: Catalyst screening for the cycloaddition of 2-methylaziridine with CO_2_; Table S2: Comparison of MCM-41@ILLaCl4 catalyst with other catalysts for the synthesis of 2-oxazolidinones; Table S3: BET surface area and pore volume of MCM-41@ILLaCl4 and MCM-41.

## Sample Availability

Samples of the compounds are available from the authors.
