# Supplementary material for: Novel MCM-41 Supported Dicationic Imidazolium Ionic Liquids Catalyzed Greener and Efficient Regioselective Synthesis of 2-Oxazolidinones from Aziridines and Carbon Dioxide
Source: Molecules. 2022 Dec 28;28(1):242. doi: 10.3390/molecules28010242 (PMC9822182; doi:10.3390/molecules28010242)
Supplement: Supplementary file 1 [file molecules-28-00242-s001.zip › molecules-2077634-supplementary.pdf]

Supporting Information

Communication

Novel MCM-41 Supported Dicationic Imidazolium Ionic Liquids Catalyzed Greener and Efficient Regioselective Synthesis of 2-Oxazolidinones from Aziridines and Carbon Dioxide

Yulin Hu [1,*], Lili Yang [1] and Xiaobing Liu [2,*]

[1] College of Chemistry and Chemical Engineering, Anshun University, Anshun 561000, China
[2] College of Chemistry and Chemical Engineering, Jinggangshan University, Ji'an 343009, China
* Correspondence: ylhanshun@126.com (Y.H.); liuxiaobing805@163.com (X.L.)

Materials and Methods

Materials and apparatus

The solvents were of analytical grade, and all chemicals were obtained from Sigma-Aldrich and used without further purification. The chemical structures of catalysts were characterized by FT-IR spectra (Nicolet Nexus 470) spectrometer. The Powder X-ray diffraction (XRD) patterns were recorded on a Rigaku Ultima IV diffractometer. Scanning electron microscopy (SEM) and Energy dispersive X-ray (EDX) analysis were performed with a JSM-7500F instrument. UV-Vis measurement was recorded on a Shimadzu UV-2450 spectrometer. N_2 adsorption-desorption measurements were measured on a Micromeritics-2010 apparatus. Thermal gravimetric analysis (TGA) was performed using a Netzsch Thermoanalyzer STA 449 analyzer under nitrogen atmosphere. ^{1}H NMR spectra were recorded on a Bruker 400 MHz spectrometer. Elemental analysis was performed on a Vario Micro cube Elemental Analyzer.

Preparation of supported ionic liquids

MCM-41 support was prepared following the reported methods.[39-41] The supported ionic liquids were prepared following the reported procedures,[24,25,43-45] and the concise route was outlined in Scheme S1. For materials, methods and characterization of supported ionic liquids are provided in supporting information. To a round-bottomed flask were added sodium ethoxide (0.4 mol), imidazole (0.4 mol) and ethanol (150 mL). After this, the mixture was vigorously stirred at 70 ^{o}C for 8 h, then 1,4-dichlorobutane (0.2 mol) was added to the mixture and stirred at 70 ^{o}C under nitrogen atmosphere for another 8 h. After that, the suspension was filtrated and the filtrate was evaporated to about 15 mL, the white solid was filtered and washed with ethanol/water solution (v/v=1/1, 10 mL × 3) followed by vacuum drying at 50 °C to afford **I**. Subsequently, (3-chloropropyl) triethoxysilane (0.3 mol) and **I** (0.15 mol) were added to the solution of dry toluene (100 mL), and the mixture was stirred under nitrogen atmosphere at 110 ^{o}C for 24 h. After that, the solvent was isolated by liquid-liquid separation and the residue was washed with ether followed by vacuum drying at 80 °C to give **II**. Afterward, $LaCl_3$ (0.2 mol) or CH_3COONa (0.2 mol) or $NaBF_4$ (0.2 mol), and **II** (0.1 mol) were added into 40 mL methanol and was stirred vigorously at 50 °C for 24 h. Then the suspension was filtered and dried under vacuum at 50 °C for 2 h to afford the anionic functionalized ILs ILanion **III**. Finally, MCM-41 (1.0 g) and ILanion **III** (0.3 g) were mixed with 50 mL anhydrous toluene in a 100 mL round bottomed flask, and the reaction mixture was stirred at 110 °C for 24 h. After filtration and drying in a vacuum to give the supported ionic liquids MCM-41@ILanion.

Catalytic synthesis of 2-oxazolidinones

Aziridine (10 mmol), and MCM-41@ILLaCl_4 (0.2 g) were added into a 20 mL stainless-steel reactor. In order to ensure the complete CO_2 environment in stainless autoclave, CO_2 was introduced and discharged into the gas cylinder three times. After that, the pressure of CO_2 was adjusted to 0.7 MPa, and the temperature probe was inserted into the temperature probe hole in the reaction kettle to adjust the reactor temperature to 50 ℃, and the reaction was carried out with constant stirring. The reaction progress was determined by gas chromatography (GC). After completion of the reaction, the reactor was moved to an ice bath to cool down for 10 min, and then CO_2 was slowly degassed. The product was then dissolved in dichloromethane, the insoluble solid catalyst was easily recovered by centrifugation and recycled directly for the next runs under identical reaction conditions. The filtrate was concentrated in vacuum to provide pure products. The obtained products are known and commercial, and were verified by comparison with those of standard compounds or by ^{1}H NMR and elemental analysis.

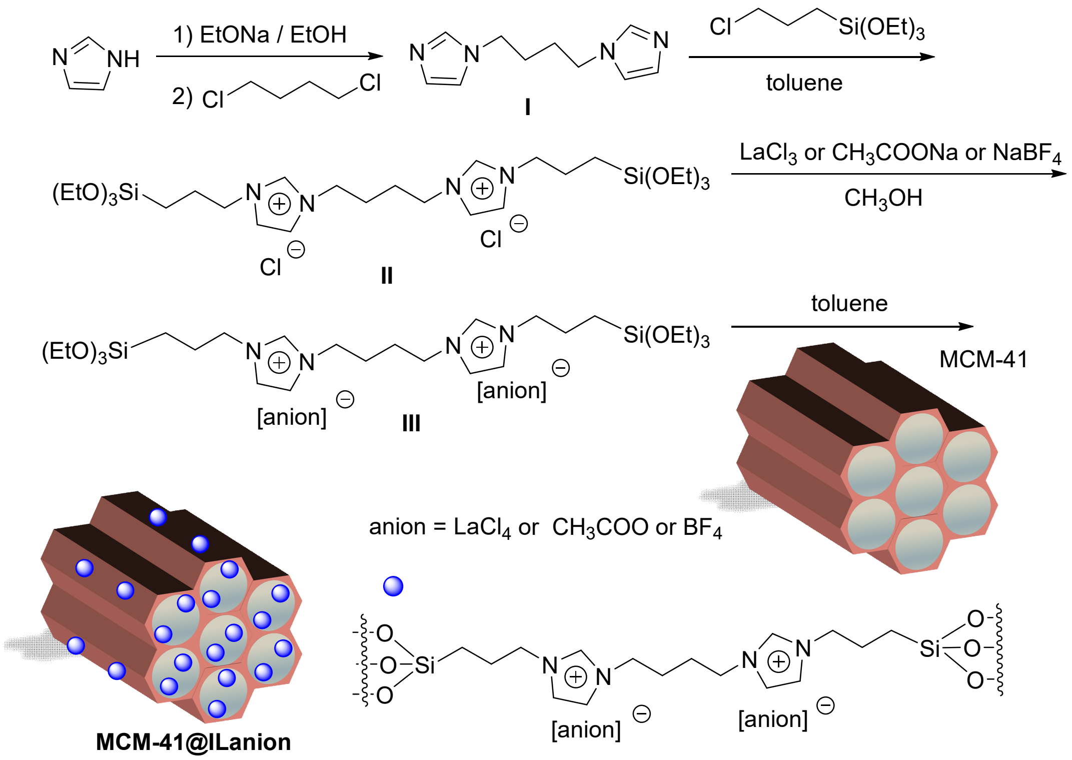

Scheme S1. Schematic paths for synthesis of supported ionic liquids.

Spectroscopic data for products

5-Methyloxazolidin-2-one (Table 2, entry 1): ^{1}H NMR (400 MHz, $CDCl_3$) (δ/ppm): 1.39 (d, CH_3, 3H), 3.15 (dd, CH_2, 2H), 4.81 (m, CH, 1H), 6.23 (s, NH, 1H); Elemental analysis for $C_4H_7NO_2$: C, 47.46; H, 6.93; N, 13.82; O, 31.61. Found: C, 47.52; H, 6.98; N, 13.85; O, 31.65.

Oxazolidin-2-one (Table 2, entry 2): ^{1}H NMR (400 MHz, $CDCl_3$) (δ/ppm): 3.16 (t, CH_2, 2H), 4.73 (t, CH_2, 2H), 6.29 (s, NH, 1H); Anal. Calcd. for $C_3H_5NO_2$: C, 41.33; H, 5.74; N, 16.05; O, 36.72. Found: C, 41.38; H, 5.79; N, 16.09; O, 36.75.

4,5-Dimethyloxazolidin-2-one (Table 2, entry 3): ^{1}H NMR (400 MHz, $CDCl_3$) (δ/ppm): 1.25-1.37 (m, $2CH_3$, 6H), 4.76 (m, CH, 1H), 5.21 (m, CH, 1H), 6.27 (s, NH, 1H); Anal. Calcd. for $C_5H_9NO_2$: C, 52.11; H, 7.84; N, 12.13; O, 27.75. Found: C, 52.16; H, 7.88; N, 12.17; O, 27.79.

Hexahydrobenzo[d]oxazol-2(3H)-one (Table 2, entry 4): ^{1}H NMR (400 MHz, $CDCl_3$) (δ/ppm): 1.28-1.45 (m, CH_2, 2H), 1.52-1.58 (m, CH_2, 2H), 1.62-1.76 (m, CH_2, 2H), 1.83-1.98 (m, CH_2, 2H), 3.75-3.77 (m, CH, 1H), 4.62 (m, CH, 1H), 6.38 (s, NH, 1H); Elemental analysis for $C_7H_{11}NO_2$: C, 59.51; H, 7.82; N, 9.88; O, 22.64. Found C, 59.56; H, 7.85; N, 9.92; O, 22.67.

5-Phenyloxazolidin-2-one (Table 2, entry 5): ^{1}H NMR (400 MHz, $CDCl_3$) (δ/ppm): 3.55 (d, CH_2, 2H), 5.62 (t, CH, 1H), 6.46 (s, NH, 1H), 7.30-7.46 (m, Ar-H, 5H); Elemental analysis for $C_9H_9NO_2$: C, 66.21; H, 5.54; N, 8.53; O, 19.55. Found C, 66.25; H, 5.56; N, 8.58; O, 19.61.

3-Methyloxazolidin-2-one (Table 2, entry 6): ^{1}H NMR (400 MHz, $CDCl_3$) (δ/ppm): 3.19 (s, CH_3, 3H), 3.62 (t, CH_2, 2H), 4.35 (t, CH_2, 2H); Elemental analysis for $C_4H_7NO_2$: C, 47.46; H, 6.95; N, 13.80; O, 31.62. Found C, 47.52; H, 6.98; N, 13.85; O, 31.65.

3-Ethyl-5-phenyloxazolidin-2-one (Table 2, entry 7): ^{1}H NMR (400 MHz, $CDCl_3$) (δ/ppm): 1.17 (t, CH_3, 3H), 3.16 (m, CH_2, 2H), 3.95 (t, CH_2, 2H), 5.45 (m, CH, 1H), 7.32-7.64 (m, Ar-H, 5H); Elemental analysis for $C_{11}H_{13}NO_2$: C, 69.05; H, 6.82; N, 7.28; O, 16.71. Found C, 69.09; H, 6.85; N, 7.32; O, 16.73.

Supported ILs characterization

XRD patterns of mesoporous support and supported ILs are shown in Figure S1. All samples produce a broad diffraction peak at around 2θ = 21-25°, suggesting the presence of typical crystalline nature of mesoporous silica structure [38-40]. The effect of IL species on the structure properties in these nanocomposites can also be observed, and the intensities of the typical peaks decreased gradually after their immobilization on the support framework (Figure S1a-c). In addition, no new diffraction peaks of IL species can be caught, declaring a well dispersion of IL on the mesoporous support.

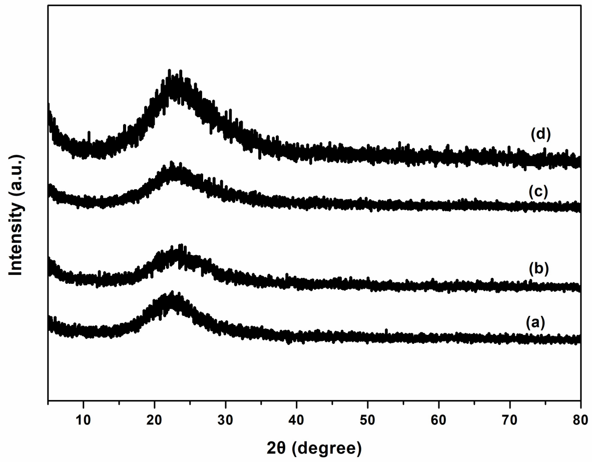

Figure S1. XRD diffractograms of MCM-41@ILBF$_4$ (a), MCM-41@ILLaCl$_4$ (b), MCM-41@ILCH$_3$COO (c), MCM-41 (d).

The surface morphologies of these nanocomposites were obtained using SEM analysis (Figure S2). The pure MCM-41 revealed a characteristic lamellar-like mesoporous morphology (Figure S2d) [38,39]. When ionic liquid was loaded onto MCM-41, supported ILs presented irregular shapes consisting of some spherical agglomerates with wrinkled nano-sized particles (Figure S2a-c). SEM images showed that the whole morphologies and well-ordered structure are retained upon the immobilization of ionic liquid on the MCM-41 support. The observed agglomeration in the SEM images is due to the successful immobilization of ionic liquid particles onto the surface of mesoporous support. The EDX analysis was also used to investigate the existing elements in the supported ionic liquids. The presence of the uniform distribution of C, Si, O, N, F, Cl, or La elements is clearly matched with the structures in these nanocomposites (Figure S3), confirming that dual imidazolium ionic liquids are successfully immobilized on the support framework.

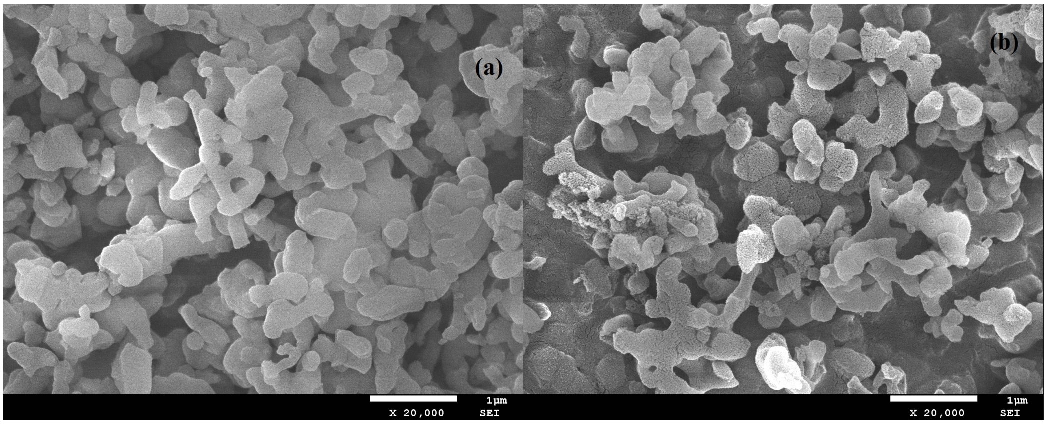

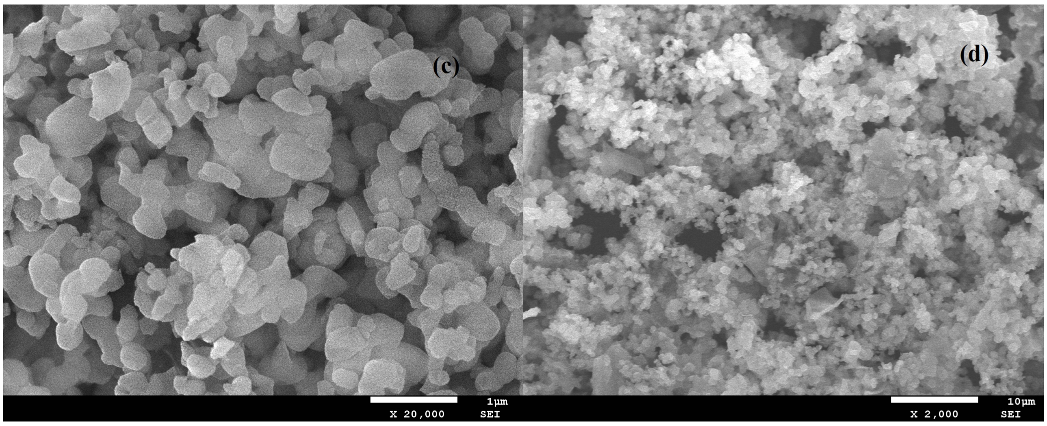

Figure S2. SEM images of MCM-41@ILBF$_4$ (a), MCM-41@ILLaCl$_4$ (b), MCM-41@ILCH$_3$COO (c), MCM-41 (d).

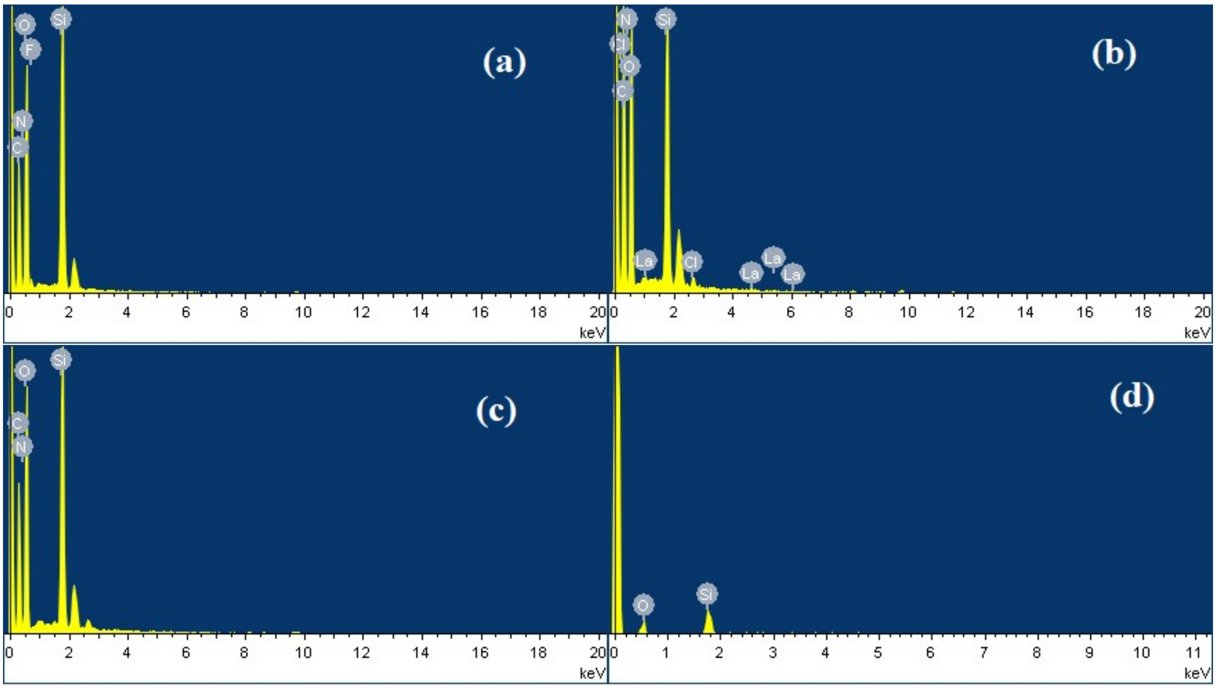

Figure S3. EDX images of MCM-41@ILBF$_4$ (a), MCM-41@ILLaCl$_4$ (b), MCM-41@ILCH$_3$COO (c), MCM-41 (d).

FT-IR spectra of the supported ILs are shown in Figure S4. The peaks at around 1075 cm^{-1}, 812 cm^{-1} are attributed to the Si-O-Si stretching vibrations. The small peaks at around 3550-3350 cm^{-1}, 962 cm^{-1} are assigned to the Si-OH stretching vibrations. The peaks at around 1621 cm^{-1}, and 1153 cm^{-1} are observed due to the characteristic peak of C=C, and C–N stretching vibration of imidazole ring, respectively [24,42-44]. The peaks at around 2938-2834 cm^{-1} are assigned to the C-H stretching vibration of methylene, and the vibration peak at around 722 cm^{-1} confirms the successful introduction of methylene groups. These results indicated that the successful immobilization of the dual imidazolium ionic liquids onto the mesoporous support. The UV–vis spectra of the samples are shown in Figure S5. The broad band centered at 225-238 nm is due to the presence of Si-O species, other peaks of Figure S5a-c located at around 320-335 nm was due to the presence of imidazole cation ring [24,43], suggesting that dual imidazolium ionic liquids are successfully immobilized on the support. N_2 adsorption and desorption isotherms and pore size distribution of the superior supported catalyst MCM-41@ILLaCl$_4$ and MCM-41 support are depicted in Figure S6. N_2 adsorption-desorption isotherms of the nanomaterials are type IV isotherms with H1 hysteresis loop at relative pressure (P/P_0) = 0.2-0.5, confirming their mesoporous structures. According to the results of specific surface area and pore volume in Table S3, the specific surface area of MCM-41 was 811.16 m^2/g, the total pore volume was 0.82 cm^3/g

and the diameter pore was 5.61 nm. After immobilization of the dual imidazolium ionic liquid, the specific surface area of MCM-41@ILLaCl4 was reduced to 412.57 m^2/g, the pore volume and pore size of the catalyst decreased to 0.38 cm^3/g, 5.23 nm, respectively, which is reason for the presence of sizes of functional ionic liquid groups in the support.

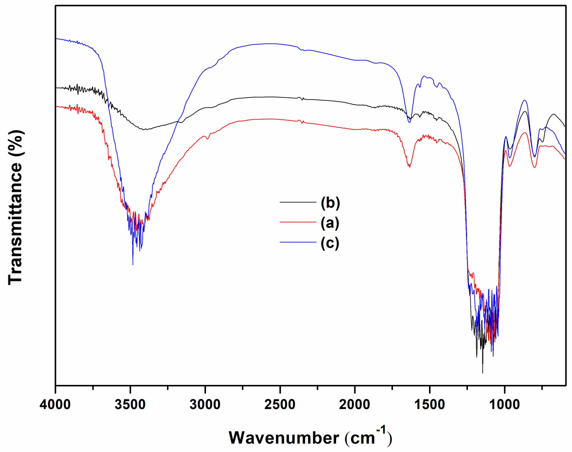

Figure S4. FT-IR spectras of MCM-41@ILBF4 (a), MCM-41@ILLaCl4 (b), MCM-41@ILCH3COO (c).

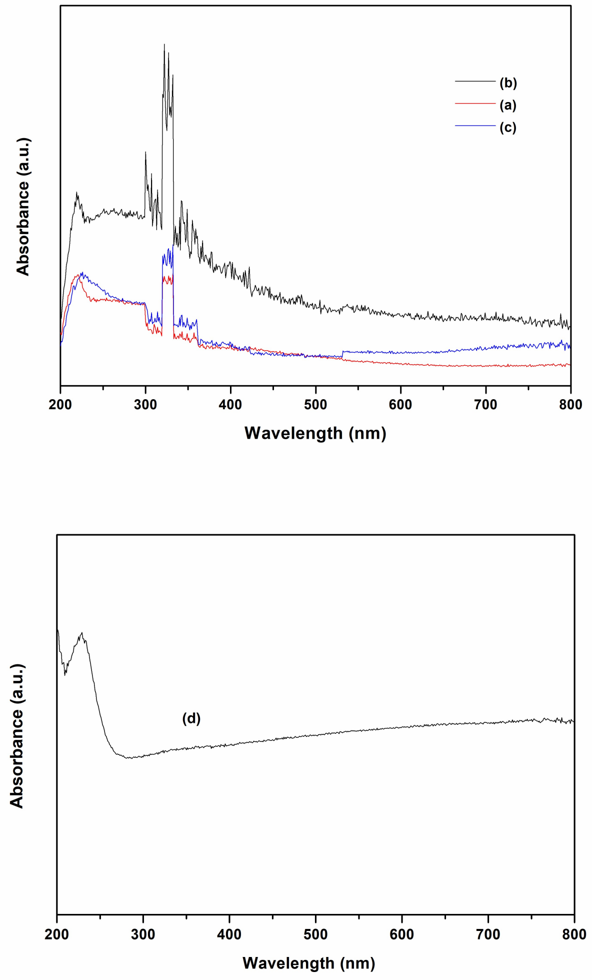

Figure S5. UV–Vis spectras of MCM-41@ILBF4 (a), MCM-41@ILLaCl4 (b), MCM-41@ILCH3COO (c), MCM-41 (d).

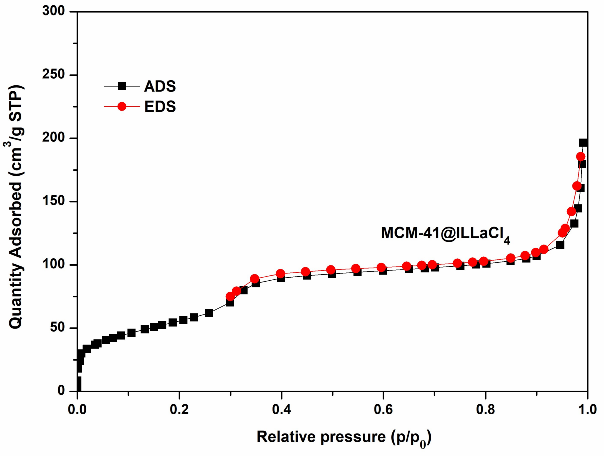
Quantity Adsorbed (cm3/g STP)
Relative pressure (p/p0)
ADS
EDS
MCM-41@ILLaCl4

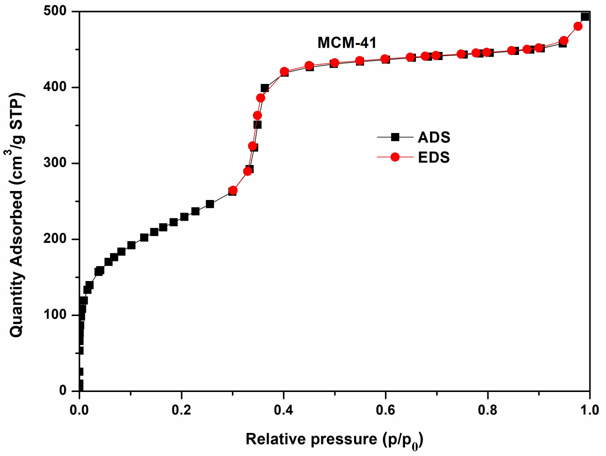
Quantity Adsorbed (cm3/g STP)
Relative pressure (p/p0)
MCM-41
ADS
EDS

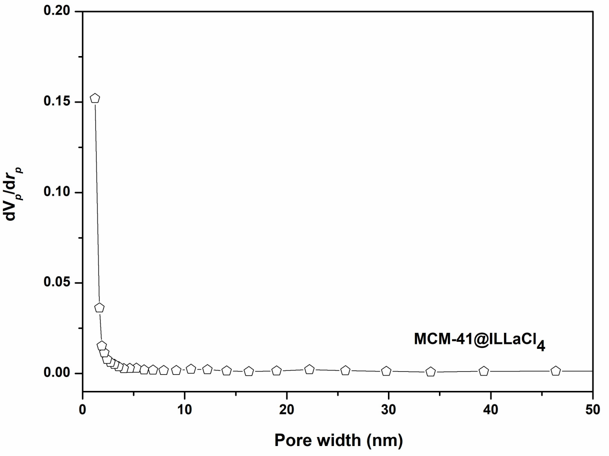
dVp/drp
Pore width (nm)
MCM-41@ILLaCl4

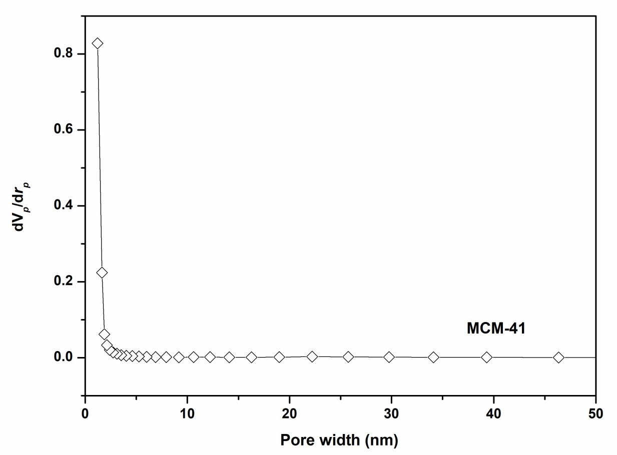

Figure S6. N_2 adsorption-desorption isotherms and pore size distributions of MCM-41@ILLaCl$_4$ and MCM-41.

Table S1. Catalyst screening for the cycloaddition of 2-methylaziridine with CO_2.[a]

Entry	Catalyst	Time (h)	Yield (%)[b]	Selectivity (%)[c]
1	MCM-41@ILBF$_4$	8	52	84.7
2	MCM-41@ILLaCl$_4$	3	95	99.3
3	MCM-41@ILCH$_3$COO	8	78	93.1
4	ILBF$_4$	6	32	75.1
5	ILLaCl$_4$	4	83	94.2
6	ILCH$_3$COO	6	73	84.5
7	MCM-41	24	15	90.4[d]

[a] Reaction conditions: 2-methylaziridine (10 mmol), CO_2 (0.7 MPa), catalyst (5%, weight percent based on the 2-methylaziridine), 50 °C. [b] Isolated yield. [c] GC analysis. [d] The reaction was carried out at 90 °C.

Table S2. Comparison of MCM-41@ILLaCl4 catalyst with other catalysts for the synthesis of 2-oxazolidinones

Entry	Catalyst	Conditions	Time	Yield/ Selectivity (%)	Ref.
1	2,2',2"-terpyridine	N-hydroxyethylaziridine, CO_2 (3 MPa), methanol, 110 °C	20 h	71/-	10
2	hierarchical porous silica	N-ethylaziridine, CO_2 (4 MPa), H_2O, 100 °C	6 h	84/97	12
3	$[NbCl_3(O_2CNEt_2)_2]$	1-methyl-2-phenylaziridine, CO_2 (1 atm), $[NBu_4]I$, 25 °C	24 h	86/-	15
4	2,3-DhaTph COF	1-methyl-2-phenylaziridine, CO_2 (2 MPa), TBAI, 50 °C	3 h	96/97	16
5	TpBpy-Cu-14	1-ethyl-2-phenylaziridine, CO_2 (1 MPa), TBAB, 30 °C	10 h	99/99	17
6	MOF containing 24-nuclear zinc nanocages	1-ethyl-2-phenylaziridine, CO_2 (2 MPa), TBAB, 70 °C	12 h	97/98	18
7	$\{tBuC_6H_4CONC_6H_3(^iPr)_2Eu[N(SiMe_3)_2]THF\}_2$	N-ethyl-2-phenylaziridine, CO_2 (1 atm), DBU, 50 °C	48 h	92/99	22
8	MCM-41@ILLaCl4	2-methylaziridine, CO_2 (0.7 MPa), 50 °C	3 h	95/99.3	This work

Table S3. BET surface area and pore volume of MCM-41@ILLaCl_4 and MCM-41

Sample	A_{BET} (m^2/g)[a]	V_P (cm^3/g)[b]	d_{pBJH} (nm)[c]
MCM-41	811.16	0.82	5.61
MCM-41@ILLaCl_4	412.57	0.38	5.23

[a] Specific surface area obtained using a Brunauer-Emmett-Teller (BET) plot. [b] Specific pore volume. [c] Pore diameter obtained using the Barrett-Joyner-Halenda method.
